# Long-term dietary intervention trials: critical issues and challenges

**DOI:** 10.1186/1745-6215-13-111

**Published:** 2012-07-20

**Authors:** Georgina E Crichton, Peter RC Howe, Jonathan D Buckley, Alison M Coates, Karen J Murphy, Janet Bryan

**Affiliations:** 1 Nutritional Physiology Research Centre, University of South Australia, GPO Box 2471, Adelaide, South Australia, 5001, Australia; 2 School of Psychology, Social Work and Social Policy, University of South Australia, GPO Box 2471, Adelaide, South Australia, 5001, Australia

**Keywords:** Dietary intervention trial, Attrition, Adherence

## Abstract

**Background:**

There are many challenges involved in running randomised controlled dietary intervention trials that investigate health outcomes. The aim of this paper was to evaluate the recruitment process, retention of participants and challenges faced in our dairy intervention trial, and to provide strategies to combat the difficulties of running long-term dietary intervention trials.

**Methods:**

A 12-month, randomised, two-way crossover study was conducted in overweight adults with habitually low dairy food consumption to assess the effects of a high dairy intake (4 servings of reduced-fat dairy per day) compared with a low dairy intake (1 serving of reduced-fat dairy per day) on measures of cardiometabolic and cognitive health. On completion of the high dairy intake phase, each participant was interviewed about their experience in the trial and responses were used to evaluate the key issues for study participants.

**Results:**

Although the recruitment target was achieved, high rates of attrition (49.3%) and difficulties maintaining participant compliance (reported by 37.8% of participants) were major threats to the viability of the study. Factors that contributed to the high attrition included inability to comply with the dietary requirements of the study protocol (27.0%), health problems or medication changes (24.3%) and time commitment (10.8%).

**Conclusion:**

Attrition and adherence to study requirements present challenges to trials requiring longer-term dietary change. Including a run-in period to further assess the motivation, commitment and availability of participants, maintaining regular contact with participants during control phases, minimising time commitment, providing flexibility with dietary requirements, facilitating positive experiences, and stringent monitoring of diet are some key recommendations for future dietary intervention trials.

**Trial registration:**

Australia and New Zealand Clinical Trials Registry (ACTRN 12608000538347)

## Background

This 12-month dietary intervention trial was designed to assess the effects of a high intake of reduced-fat dairy food on cardiometabolic and cognitive health in overweight, habitually low dairy consumers. The difficulties of running longitudinal studies and randomised controlled trials to investigate health outcomes are well recognised [1-4]. Implementing a dietary intervention to assess physical and psychological outcomes of increased dairy consumption presented multiple challenges, including recruitment of interested volunteers and maintaining subject compliance, both critical for the success of any health research. One of the main challenges presented by the nature of this investigation is the need to conduct a long-term evaluation (that is, 6 months) and the relative benefit of doubling this time in order to conduct a crossover that will more than halve the subject requirement.

The purpose of this article is not to present the results of the study [5,6], but to indicate some of the difficulties faced, barriers to completion and challenges of running longer-term dietary intervention trials. Previous research examining the challenges of running randomised controlled trials and evaluating health outcomes have recommended the inclusion of a behavioural run-in period prior to randomisation [1,2], minimising the time between obtaining consent and participation [4], including a lifestyle modification component to the intervention [1], targeting more men during recruitment [1], and contacting potential participants directly [3]. This study extends previous research by offering suggestions specific to dietary intervention trials, based on experiences in the present trial, to enhance compliance and minimise attrition. This should assist researchers in their efforts to successfully implement and assess the effects of similar studies in the future.

## Methods

### Study design

A 12-month, randomised, two-way crossover dietary intervention trial was conducted in Adelaide, South Australia at the Nutritional Physiology Research Centre(NPRC). The key aims of the study were to assess the effects of a high intake of reduced-fat dairy food on cardiometabolic health and cognitive function [5,6]. Overweight, habitually low dairy consumers were randomised to either the high dairy (HD) group (4 servings of reduced-fat dairy per day) or the low dairy (LD) group (1 serving of reduced-fat dairy per day). A crossover design was implemented to allow comparison of the LD and HD diets within the same individual. Participants act as their own controls in crossover studies so individual differences are controlled for, making the error variance smaller and subsequently reducing the sample size required to find a significant effect due to increased statistical power [7]. This design was also adopted in an effort to minimise attrition and to maximise participant interest and compliance by enabling each participant to experience both diet conditions and receive complementary dairy food. Individuals switched to the alternate diet after 6 months. Ethical approval was obtained from the University of South Australia Human Ethics Committee.

### Participants and recruitment

Participants were overweight or obese adults aged 18 to 75 years who had a self-reported habitually low intake of dairy (<2 servings per day), selected in line with the average dairy consumption of the Australian population [8]. Overweight or obese people (body mass index ≥25 kg/m^2^) were chosen as the target population to assess the primary outcome measure (waist circumference) and also because obesity is associated with a greater risk of cognitive decline [9,10]. Participants also had to be able and willing to attend the research centre for testing at baseline and at 6 and 12 months, and be willing to collect dairy weekly for 6 months from the centre while in the HD arm.

Exclusion criteria included being a current smoker, body weight exceeding 135 kg (maximum capacity of the dual energy X-ray absorptiometry), diagnosed with diabetes, cardiovascular disease, liver disease, renal disease or stage 2 hypertension (>160/100 mm Hg), and pregnancy or the possibility of pregnancy within 12 months. Consumption of more than 1 g of fish oil per day, regular use of appetite suppressants, weight loss medications, or any other medication that may have influenced the study outcomes prevented inclusion. Participants were excluded if they had a known allergy or intolerance to dairy or lactose, or were considered unlikely to comply with the study protocol.

Multiple strategies were employed to maximise recruitment. Advertisements seeking people to participate in a trial examining the health benefits of dairy were placed in a local newspaper. Similar written advertisements were placed on noticeboards around the university and in several public places (local hospital, libraries and shopping centres). During the recruitment period, a short interview segment on a current affairs programme was shown on local television promoting the study and discussing the possible health benefits of dairy, including weight loss.

Interested potential volunteers were invited to an information session and pre-study screening, in which some simple health measures were taken (height, weight, blood pressure) and health and dietary questionnaires were completed to determine eligibility for inclusion in the study. Potential participants were required to stipulate the typical amount, type and brand of milk, yogurt, cheese, custard, ice-cream, cream and butter they consumed in an average week.

All volunteers understood the dietary and exercise requirements of the study, and gave informed written consent if deemed eligible for inclusion. Thirty-six volunteers were randomised into the HD group, and 35 were randomised into the LD group. All volunteers were offered monetary compensation of $200 upon completion of the study for expenses incurred in travelling to the research centre for testing.

### Dairy intervention

Participants randomised to the HD diet were required to incorporate 4 servings of reduced-fat dairy per day into their diet. They were required to visit the research centre weekly (or fortnightly if unable to come in weekly) to collect 28 servings of dairy provided to them each week. To ensure the dairy products remained chilled, ice-bricks and cooler bags were provided to aid with transporting the dairy foods from the research centre. Dairy products included a selection of reduced-fat milk, yogurt and custard, and were provided based on personal preference. All participants were instructed on how to substitute other foods for dairy so as not to increase their overall energy intake.

During the LD phase, participants were instructed to continue their usual diet, but to limit their dairy intake to no more than 1 serving per day. Dairy products were not provided during this phase of the study as this level of intake reflected participants’ typical habitual intake. During the LD phase, participants attended the clinic only for assessments (at baseline and at the end of each dietary phase). All participants were instructed to maintain their normal physical activity for the duration of the study.

All participants were provided with verbal and written instructions on the quantities of different dairy foods that constitute 1 serving (for example, 250 ml milk, 175 to 200 g yogurt, 190 g custard). Compliance during the HD phase was measured through the completion of dairy logs, whereby participants recorded all dairy they consumed on a daily basis. Weight was measured fortnightly; if weight gain was noticed, participants were offered a time to speak with a registered nutritionist to discuss ways of incorporating the dairy into their diet. The aim of the nutritional counselling was to assist the participant in successfully incorporating the dairy into their diet by substituting it for other foods. The aim was not to enforce a particular diet but rather to provide suggestions allowing the volunteer to make choices on how to incorporate the dairy.

All participants were sent letters from the research team in the 2 weeks preceding an assessment to remind them of their upcoming visit. Any details about specific requirements (for example, fasting the night before) were given. Reminder telephone calls were made to each participant in the week prior to their scheduled appointment to confirm suitability. If participants did not attend a scheduled appointment, a maximum of three telephone calls were made and one letter (or email) sent prior to withdrawing the participant from the study. Participants were given every opportunity to either telephone and reschedule, telephone and discuss their concerns, or withdraw from the study.

### Outcome measures

Participants had fasting clinic assessments conducted over two consecutive mornings at baseline and at 6 and 12 months. The total testing time was approximately 3.5 hours. Water was permitted on the morning of testing but participants were instructed not to undertake any physical activity prior to testing. Diet and physical activity questionnaires (3-day weighed food records, 3-day physical activity diaries, food frequency questionnaires) were completed at each assessment.

Blood pressure, anthropometry and biochemistry including blood samples were measured at each clinic assessment [5]. Cardiometabolic outcomes included body weight, waist circumference, percentage total and abdominal body fat (dual-energy X-ray absorptiometry), systolic and diastolic blood pressure, fasting plasma glucose, triglycerides, high-density lipoprotein, low-density lipoprotein and total cholesterol, resting metabolic rate (by indirect calorimetry), and arterial compliance (pulse wave velocity). The cognitive assessment consisted of a battery of 10 neuropsychological tests, a depression, anxiety and stress questionnaire, and one measure of pre-morbid intelligence [6]. A wide range of cognitive abilities were assessed, including information-processing speed, attention, memory, verbal functions and language, concept formation and reasoning, executive function, and perception. The cognitive testing component took approximately 1 to 1.5 hours to complete.

### Participant feedback

**Figure 1:**
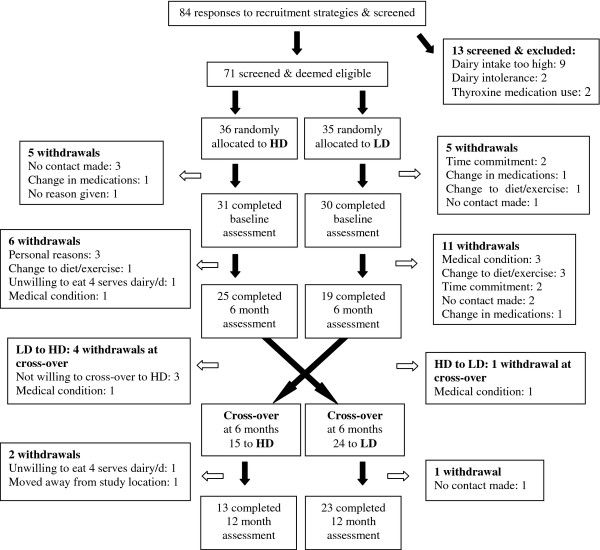
**Participant flow and attrition.** Study design, randomisation to the high dairy (HD) and low dairy (LD) diets, and participant flow through the study, including reasons for withdrawal at all stages.

The high number of participants who dropped out of the study (outlined in Figure 1) made it apparent that participants were finding the trial challenging and that study completion with adequate power would be in doubt. Consequently, in order to gain constructive and honest participant feedback about the trial, a 15-item, face-to-face open-ended interview (Appendix A) was added to the study and conducted with each participant who completed 6 months on the HD diet at the end of this phase. The questions were based around the following broader topics: dairy and food, dietary habits, support/learning, and practical considerations of study involvement. Examples of questions asked included ‘did you learn anything about your dietary patterns or eating habits while on the high dairy diet?’ and ‘what was the most difficult thing about being in the trial?’ Interviews were conducted with only 37 participants (11 males and 26 females) and took between 10 and 20 minutes to complete. Thirty-six of the 37 participants completed the 12-month trial.

### Data analysis

Based on waist circumference, the primary outcome measure, a total sample of 34 participants was estimated to give 80% power to detect an effect size of 0.5 (predicted change/standard deviation of change) at an α value of 0.05 [11]. Statistical analyses were performed with SPSS for Windows, version 18.0 (SPSS Inc., Chicago, IL, USA). Analysis of variance and chi-square tests were used to determine any differences in baseline demographic, cardiometabolic or dietary variables between those who completed the entire 12 months of the study (completers, *n* = 36), those who completed part of the study but withdrew prior to completion (late drop-outs, *n* = 25), and those who were screened and enrolled but withdrew before commencement of the intervention (early drop-outs, *n* = 10).

## Results

### Recruitment and retention

Eighty-four potential participants expressed an interest in participating and were screened. The most interest for participation was generated from a segment shown on television about the health benefits of dairy (35.7% of respondents), from an advertisement in the local newspaper (31%), and from participants who had previously participated in studies at the research centre and had indicated an interest in being contacted for future studies (16.7%). Of the 84 people who were initially screened, 71 were eligible to participate, gave consent and were enrolled and allocated to treatment. Sixty-one participants (18 male, 43 female), completed a baseline assessment. A further 25 participants withdrew from the study after completing a baseline assessment. A significantly larger number of participants who were initially randomised to the LD diet, compared with the HD diet, withdrew from the study (22 compared with 13; *P* <0.05). In total, 36 adults (10 males, and 26 females) aged 18 to 71 years completed the 12-month study. The overall drop-out rate was 49.3%. Figure 1 outlines the number of participants recruited and tested at each time point, and the reasons for withdrawal.

The most frequent reasons cited for leaving the study were an inability to adhere to the dietary or physical activity requirements of the study (27.0%), changes the participant intended to make that would interfere with study outcomes, such as starting new medication or having a medical condition revealed during the course of the study (24.3%), and time commitment (10.8%).

Although there were more females (*n* = 51) than males (*n* = 20) in the study, there was not a significant difference in drop-outs in terms of gender (Table 1). Completers had significantly lower percentage abdominal fat at baseline than drop-outs (*P* <0.05). There were no other significant differences between completers and noncompleters.

**Table 1:** Demographic, cardiometabolic and dietary characteristics of the total sample at baseline

**Demographic variable**	**Early drop-outs**^a^**(*n*** **= 10)**	**Drop-outs during study (*n*** **= 25)**	**Completers (*n*** **= 36)**	***P*value**^**b**^
Initial diet allocation				
High dairy	5 (7.0)	8 (11.3)	23 (32.4)	0.013^c^
Low dairy	5 (7.0)	17 (23.9)	13 (18.3)	0.013^c^
Gender				
Male	2 (10.0)	8 (40.0)	10 (50.0)	NS
Female	8 (15.7)	17 (33.3)	26 (51.0)	NS
Presence of medical conditions	3 (30)	7 (28)	13 (36)	NS
Taking medications	6 (60)	9 (36)	13 (36)	NS
Alcohol consumption (>2×/week)	1 (10)	5 (20)	8 (22)	NS
Physical activity (≥3×/week)	8 (80)	20 (80)	32 (89)	NS
Age (years)	44 (12)	45 (16)	49 (14)	NS
Weight (kg)	95 (15)	93 (19)	89 (17)	NS
Waist circumference (cm)	NA	101 (14)	98 (14)	NS
Body mass index (kg/m^2^)	36 (6)	33 (5)	32 (6)	NS
Total body fat (%)	NA	45 (7)	43 (9)	NS
Abdominal fat (%)	NA	47 (6)	43 (7)	0.043
Systolic BP (mmHg)	125 (11)	132 (1)	127 (14)	NS
Diastolic BP (mmHg)	75 (8)	78 (11)	74 (11)	NS
Total psychological well-being (DASS)^d^	NA	18 (17)	14 (19)	NS
Depression	NA	5.3 (7.9)	3.9 (6.2)	NS
Anxiety	NA	3.8 (4.0)	3.2 (5.9)	NS
Stress	NA	9.3 (8.3)	7.1 (7.8)	NS
Energy intake (MJ/day)	NA	8.8 (2.3)	8.9 (3.2)	NS
Protein (g/day)	NA	95 (26)	96 (32)	NS
Fat (g/day)	NA	82 (30)	80 (39)	NS
Carbohydrate (g/day)	NA	219 (66)	231 (90)	NS
Alcohol (g/day)	NA	8.5 (11.1)	7.6 (12.4)	NS
Calcium (mg/day)	NA	920 (352)	1096 (764)	NS

Demographic, cardiometabolic and dietary characteristics of the total sample (*n* = 71) at baseline, comparing those who withdrew before commencement of the intervention (early drop-outs) with those who withdrew during the study and with those who completed the 12-month study. Data presented as *n* (%) or mean (standard deviation). BP, blood pressure; NA, not assessed; NS, not significant. ^a^Participants withdrew between screening and baseline assessment. ^b^Analysis of variance for continuous variables; chi-square test for categorical variables. ^c^All drop-outs grouped together (*n* = 35) and compared with completers (*n* = 36). ^d^Depression Anxiety Stress Scale (DASS) assessed only in the cognitive subgroup (drop-outs, *n* = 21; completers, *n* = 31); higher scores indicate greater depression/anxiety/stress.

There was no evidence of any period, order or seasonal effects for any of the cardiometabolic or cognitive outcomes [5,6]. Total energy intake and physical activity at the end of the two diet phases did not differ according to the order of intervention. Compliance during the HD phase was excellent; the average weekly intake of dairy was 25.2 ± 3.1 servings of the provided dairy and 2.9 ± 2.4 servings of the participant’s own dairy, giving a total intake of 28.1 ± 2.6 servings for those who completed the HD phase. Adherence to completing the diet and physical activity questionnaires was also very good, with 89% of participants completing all questionnaires at each time point.

### Participant feedback

Gaining information about aspects of the study that participants found most challenging was an important component of the interview, and was the primary reason for adding these interviews to the study (Table 2). The most frequently reported reasons for finding the study difficult were the regular visits to the research centre for dairy collections and consuming the required dairy food. The interviews did reveal some positive or beneficial aspects of participation. These included eating the dairy, meeting new people, and having health assessments.

**Table 2:** Reflection interview responses: positive and negative aspects of study participation reported by participants

**Aspect of study**	**Total number of responses**^a^
Positive	
Eating dairy	17
Meeting new people (researchers)	9
Health assessment/observing any changes in health	7
Trying something new in diet	5
Visits to the centre	4
Feel like helping	2
More aware of physical activity	2
Becoming more aware of own diet	1
Cognitive testing (interesting/enjoyable)	1
Weight loss	1
Budget (free dairy food)	1
Negative	
Coming to centre for dairy collections/traffic/parking	17
Eating the dairy	9
Monitoring/adjusting diet/having to plan meals	5
Reducing dairy intake in low dairy condition	5
Time off work/time commitment	4
Blood-taking	2
Fasting	2
Weight gain	1
Weighing food	1

Positive and negative aspects of study participation as reported by participants at the completion of the high dairy dietary phase (*n* = 37). ^a^Questions were open-ended, allowing for multiple responses from each individual.

The HD diet had a positive impact on other eating habits for some people. Many participants made some healthy changes to their diet to incorporate the dairy, such as eliminating foods high in fat or sugar and replacing them with the reduced-fat dairy. One may speculate that as a result of making dietary changes and diet monitoring by completing dairy logs and 3-monthly food records, participants became more aware of any pre-existing unhealthy eating habits. Of the 37 participants who completed the HD intervention and interview, 27 reported that at the end of the study they would be more likely to consume more dairy than they did prior to the study.

## Discussion

Two of the major challenges to any long-term study are the recruitment and retention of eligible study members for the duration of the study. While recruitment targets were met in the present study, retention was poor. Attrition is problematic as it can result in a study being statistically underpowered and can threaten external validity if it results in a more homogeneous sample than the original representative group. Attrition as a result of the intervention being studied may selectively bias the results having a detrimental effect on the internal validity of the study. The treatment effect can also be influenced as attrition may result in non-random missing data because those that withdraw do not receive the full intervention. While attrition in this crossover trial was high, it did not affect power because 34 participants were required. In addition to these direct problems, attrition also results in an inefficient expenditure of time and resources. The aim of all research involving human participants is to minimise all types of attrition in order to maintain maximum statistical power, and to minimise selection bias.

Attrition is well recognised as a common problem in health research and weight-loss studies. Socioeconomic, demographic, behavioural and health factors among participants that may predict participation or drop-out have been extensively examined. Medical conditions accompanying overweight or obesity in participants and simultaneous treatment may influence both attrition and outcome [12]. The attitudes to healthy eating and dieting in an overweight group may also be relevant factors when considering study retention, because attrition in obesity studies has been positively [13] and negatively [14] associated with binge eating, and positively and negatively associated with previous dieting [15]. Practical difficulties have been attributed as explanations for attrition in randomised controlled trials, including family or work problems, logistic difficulties such as travel and associated costs, and having to attend additional appointments [16-18]. Drop-out due to a perception that the intervention will not be of benefit, because early success is not seen, or because the participant is not randomised to their desired intervention are recognised threats to health research [16-19]. Depression [4,19,20] and low self-esteem [2] are frequently cited predictors of attrition. The findings of Fabricatore and colleagues suggest that higher baseline depression, even below levels of clinical severity, may also negatively impact upon the individual’s ability to make behavioural changes involved in weight loss [2].

### Evaluation of the trial

#### Recruitment, retention and challenges

The high attrition rate was not anticipated in the present study, particularly from within the LD group. The reasons for attrition were consistent with past research. Logistic difficulties, the health characteristics of the study population, and having unrealistic study expectations and treatment preferences were all factors that contributed to the high drop-out rate. Surprisingly, logistic difficulties appeared to be the main reason for drop-out from within the LD group, whose time commitment was considerably less than for those in the HD group. One may suspect that the anticipated upcoming time commitment during the next (HD) phase was the reason for their withdrawal.

Those participants who withdrew between screening and the final assessment were slightly younger than those who completed the study. Perhaps older individuals such as retirees may have been more compliant and willing to participate as they had more time, and enjoyed the process of coming into the research centre and having social contact with the study team. One could also hypothesise that older individuals feel more responsibility to complete something that they have commenced, or are more motivated to make a behavioural or lifestyle change to improve their health if they have experienced some degree of impairment already [2]. While considerably more women than men were enrolled in the present study, which may reflect a greater interest in health and/or diet by women, attrition was not significantly different between genders.

Adherence to the study protocol was a major challenge. One may speculate that overweight or obese people may find it particularly difficult to make a long-term dietary change, particularly in the absence of any immediate results. A lack of early success while on the HD diet, in terms of weight loss, probably contributed to the withdrawal of four participants. These volunteers put on between 0.4 and 2.3 kg in the first 6 weeks of the HD intervention. For 6 months, participants were required to make some considerable changes to their eating habits. In addition to increasing their intake of dairy, they had to ensure that their overall energy intake was not increased by incorporating the dairy rather than simply adding it to their normal diet. Participants were therefore required to not eat other foods or beverages to prevent weight gain. Although the dairy food was provided free of charge, the ability to consistently consume 4 servings per day and forgo certain other foods may have been too challenging. Reasons that emerged from the reflection interviews as to why this dietary change was difficult included the quantity of dairy that had to be consumed, the daily consumption becoming repetitive and boring, and a lack of choice of dairy foods.

The long duration of the trial certainly impacted on attrition, as participants found it difficult to maintain the increased dairy intake for 6 months. Some participants reported that planning their meals and selecting new recipes to incorporate the dairy were added burdens. Consequently, the inability to incorporate the dairy whilst still eating other food may have resulted in weight gain, frustration and subsequent drop-out.

Dietary intervention studies may be particularly challenging for depressed individuals if they eat to combat their negative feelings or other symptoms of depression. As a result they may be unable to comply with the study requirements if a strict diet is required. Depression may not only predict drop-out, but may also impact upon outcomes. The present study did not support this research. Baseline psychological well-being (depression, anxiety and stress) was assessed in the participants enrolled in the cognitive component of the study (*n* = 52 at baseline). Depression, anxiety and stress scores (and total composite score) did not differ significantly between those who completed the study from this subgroup (*n* = 31) and those who withdrew (*n* = 21). Depression scores did not significantly differ between dairy diet phases, indicating that dairy consumption did not impact depression.

### Study limitations

Total energy intake was increased during the HD period, indicating poor compliance with the instruction to substitute dairy for other foods. Expecting participants to achieve this in the present study without an individualised dietary plan is an acknowledged limitation.

The reflective interview was conducted with 37 participants, but only 36 of these completed the study. The views of those who stayed in the study versus the participant who withdrew are unable to be separated, and this information may have provided additional insights into how attrition could be minimised in future trials. Similarly, other factors that may affect attrition were not examined in the present study, such as attitudes toward food, dieting and weight loss, or motivation to change. Whether these factors may have impacted upon attrition in the present study cannot be determined.

Compliance may also have been better if a greater variety of dairy foods, as in real life, was included. Milk and yogurt were the predominant dairy foods supplied in the present study; dairy foods such as ice-cream and cheese (for example, cream cheese, cottage cheese) were not included. These dairy products were chosen because of their high whey protein content, as whey (rather than casein protein) is thought to play a role in the anti-obesity effect of dairy [21]. For example, a cheese slice on a sandwich is an easy way to incorporate dairy, but because of the primary aim of this study cheese was not included.

### Recommendations for future trials

#### *Successful recruitment and screening*

Recruitment should involve a variety of methods to attract a wide range of volunteers, of desired type and number, and thereby increase representativeness. A thorough pre-study screening session to determine the eligibility of each potential volunteer is essential, strict inclusion and exclusion criteria must be adhered to, and the study protocol and requirements should be thoroughly explained. The screening procedure should be completed in a timely manner, and the time between when consent is given and the study commences should be minimised [4]. Engaging interest early may help to prevent very early drop-outs before the study intervention begins. Further recruitment may be considered if the number of early drop-outs is high, but consideration must be given to cost and the additional time that this will add to the study.

Including a run-in period – a time after enrolment but before randomisation that allows further assessment of a participant’s eligibility and commitment to a study – may be worthy of consideration. Incorporating this time into a study enables the individual to think further about what their participation will require, and, secondly, allows the researcher to more thoroughly estimate participant compliance. Low motivation, poor commitment or limited availability may exclude a potential participant before the study commences.

#### *Flexibility with a nutritional intervention*

Offering a range of different products and brands that still meet the nutritional requirements of the study would be recommended for future dietary trials. The provision of an additional dairy option (reduced-fat cottage cheese) was made available to some participants who were finding it difficult to consume 4 servings from milk, yogurt and custard. Providing the participants with a selection of different dairy products was a way of giving them some control in this intervention study, and would be recommended in the future.

#### *Reducing time commitment*

Minimising the time commitment during any long-term trial is important. Providing alternative dairy collection points may have helped reduce this burden by providing a more convenient location to pick up the dairy, such as at grocery stores.

#### *Maintaining regular contact*

Contact with participants during a control phase when they are otherwise not actively involved in any intervention is needed. In a dietary study, control food products could be provided as a means of maintaining both contact and participant interest. This may include a selection of healthy food products such as fruit and vegetables. The additional contact may maintain the participants’ awareness and interest in both the study and their dietary patterns, and may have the added benefit of taking the focus away from the main study food so that the participants do not focus on one arm more than the other.

#### *Monitoring of compliance and nutritional counselling*

The provision of more stringent instructions on what participants are allowed to consume during an intervention phase and stricter monitoring of overall energy intake would be recommended for future trials. This could be achieved through the provision of an independent dietary plan to each participant with specific details regarding the quantity of other foods and beverages that would need to be removed from the diet in order to maintain isocaloric intake. The use of empathic staff (for example, nutritionists) to implement sound, structured dietary guidelines should be utilised. Adding a third arm to the study with the aim of weight loss (increased dairy food intake in combination with energy deficiency) may have been of benefit in terms of achieving the study aims and generating and maintaining interest in the trial.

#### *Follow-up after nonattendance*

Every endeavour should be made to contact any participant who does not attend an appointment, in the hope of rescheduling visit times or providing an opportunity for the individual to discuss why they may not want to attend.

#### *Facilitate positive aspects of trial participation*

Factors that play a positive role in the experiences of participants should be facilitated for all participants. Positive experiences such as meeting new people, visiting the research centre and trying something new in the diet should be highlighted and encouraged.

#### *Financial rewards*

Financial compensation has been demonstrated to maximise compliance [22]. Higher financial rewards may be required when a significant dietary change is required for a long duration.

## Conclusions

Improving our understanding of the risk factors for attrition either via active withdrawal or nonreturn may help to plan studies in the future to minimise attrition. This includes the identification of motivations to participate and barriers that inhibit completion. All potential participants need to have a clear understanding of what the study entails, specifically what will be required of them, and what they may realistically expect in terms of results throughout the study period in order for them to commit to the study. A run-in period may help to achieve this. Minimising the time commitment and burden required of participants at all stages is important, as is maintaining regular telephone contact with participants who are not required to attend regular appointments. This could alternatively be achieved through the provision of a control diet or food to increase involvement during the control phase. Detailed, clear and individualised dietary guidelines need to be provided to participants who are required to make a substantial change to their diet. Positive experiences of trial participation should be facilitated. Including a measure of health beliefs, attitudes toward eating and dieting history may also help to inform the characteristics of an overweight population.

Identifying and addressing any barriers to participation is an important step in minimising the problems associated with high attrition. The challenges faced and lessons learned from this study are applicable to any intervention trial in which subjects are required to make a long-term dietary change. Reducing the incidence of attrition in any long-term intervention or longitudinal research will maximise both internal and external validity and will enhance the overall quality of data collected.

## Appendix A. Reflection interview

### A.1. Dairy and food behaviours

1.  How did you find increasing your dairy intake?

2.  Was incorporating 4 serves of dairy each day easier or harder than you thought it might be?  Why?

3.  Did you find that you tended to incorporate the dairy into your diet at a specific time of day? When? (e.g., morning/middle of day/evening)

4.  What dairy food did you likea)the most?b)the least?

a) the most?

b) the least?

5.  Did being on the high dairy diet influence:a)your food or beverage choices at home?b)how you planned your other meals?c)your normal cooking habits?d)food or beverage choices if you went out to eat/drink?e)other members of your family?

a) your food or beverage choices at home?

b) how you planned your other meals?

c) your normal cooking habits?

d) food or beverage choices if you went out to eat/drink?

e) other members of your family?

6.  Do you think differently about dairy now compared to 6 months ago?

7. How do you feel now about reducing your dairy intake to less than 1 serve per day?

8. At the end of 12 months are you likely to eat more/less/or similar amounts of dairy than you did prior to your participation in this trial?

### A.2. Support/learning

9.  Have you learnt anything about yourself being in this study to date?

10.  Did you learn anything about your dietary patterns/eating habits while on the high dairy diet?

11.  Did you feel like you had adequate support:a)from the staff here? (If not, what more could we have done to help your experience?)b)from your family or friends?

a) from the staff here? (If not, what more could we have done to help your experience?)

b) from your family or friends?

## Appendix B. Practical implications

12.  What have you enjoyed the most about participating in this trial?

13.  What was the most difficult thing about being in the trial?

14.  Did it get any easier or harder as the time went on?

15.  Would you be happy to participate in any future studies at the NPRC?

16.  Is there anything else you would like to tell me about your experience?

## Abbreviations

HD, high dairy; LD, low dairy.

## Competing interests

The authors declare that they have no competing interests.

## Authors’ contributions

PRCH, KJM, JDB, AMC and JB conceived the study. All authors participated in its design and coordination. KJM and GEC collected the data. GEC performed the statistical analyses and wrote the first draft of the manuscript. PRCH, KJM, JDB, AMC and JB revised the manuscript. All authors read and approved the final manuscript.
